# Innovations and best practice in undergraduate education

**DOI:** 10.12688/f1000research.8453.1

**Published:** 2016-04-12

**Authors:** George R. Littlejohn, Graham Scott, Mary Williams

**Affiliations:** 1 School of Biosciences, College of Life and Environmental Sciences, University of Exeter, Exeter, UK; 2 School of Biological, Biomedical and Environmental Sciences, University of Hull, Hull, UK; 3 American Society of Plant Biologists, Rockville, USA

**Keywords:** Undergraduate, Education, Teaching, Learning, Biology

## Abstract

University-based scientists hold the collective responsibility for educating the next generation of citizens, scientists and voters, but the degree to which they are individually trained and rewarded for this pursuit is variable. This *F1000Research* channel has its origin in a Society for Experimental Biology Conference held in Prague, 2015 and brings together researchers who excel at undergraduate education or the scholarship of teaching and learning to discuss challenges and best practices in contemporary higher science education.

## Editorial

All experimental biologists are, to a lesser or greater extent, educators and engagement with undergraduate education is an important part of experimental biology. This *F1000Research* channel has its roots in the *Innovations and best practice in undergraduate education* session which was held at the 2015 Society for Experimental Biology (SEB) Annual Main Meeting (AMM) in Prague. This session was very well attended and notable for the high standard of oral and poster presentations, as recorded by the event’s Twitter stream ( https://storify.com/dave_thesmith/seb-2015-education-symposium).

The goal of this session and *F1000Research* channel were to highlight how teaching excellence can be achieved in undergraduate biology. The first keynote speaker, Susan Singer, Division Director in the Division of Undergraduate Education at the US National Science Foundation and a Professor at Carleton College in Minnesota, spoke about how this foundation is supporting educators in implementing effective teaching practices. The other keynote speaker, Graham Scott, a Professor at the University of Hull, described efforts to harness student enthusiasm to enhance learning. The session also included talks from SEB Section Chairs John Love and Craig Franklin, Education and Public Affairs President’s Medallist, Gonzalo Estavillo and Global Teacher of the Year Nominee, Richard Spencer. David Smith, Anne Tierney, Radka Dvorakova, Beth Dyson, Ros Gleadow, Irina Strizh and Katherine Hubbard also contributed well-received talks. There were 21 submitted abstracts and an inaugural *SEB+ Irene Manton Poster Prize* was awarded to Millie Mockford.

Several speakers from the SEB session contributed papers to this *F1000Research* channel. Smith (2016) describes how he uses 3D printed models of biological molecules to facilitate discussion of biochemical problems in large group teaching, and argues that experiential learning engendered in the students by this approach enhances the understanding they have of the objects and processes discussed in the accompanying lecture material and discussion exercises. Henri, Morrell & Scott (2015) show that essays with a question developed by students themselves in the title gained higher marks in assessments that those which didn’t. A pair of papers from Gleadow and colleagues, discuss firstly how digital technologies may be implemented to enhance student engagement and collaboration ( Gleadow *et al.*, 2015a) and then present a case study of how blended learning can be used as a teaching space, rather than simply as a repository for course material ( Gleadow *et al.*, 2015b). Lastly, Tierney (2016) discussed the importance of research-focused and teaching-focused academics coming together as a community to further the development of excellence in undergraduate teaching and learning.

The opportunity to discuss and share effective pedagogies whilst attending a primarily research-focused conference clearly resonates both with those who describe themselves primarily as researchers who teach and those whose remit of mainly teaching and learning. Several attendees at the SEB Education session held in 2015 reported, both via an SEB questionnaire and verbally to committee members and SEB staff at the AMM, that attendance at the education session was the primary reason they attended the AMM. In light of this, a similar session is scheduled for the SEB AMM to be held in 2016, with the opportunity for additional papers to be added to this channel.
